# Interobserver Agreement of Post-operative Perth Computed Tomography Protocol Data in Total Knee Arthroplasty

**DOI:** 10.7759/cureus.34349

**Published:** 2023-01-29

**Authors:** Lynee C Jones, Jil A Wood, Samuel J MacDessi

**Affiliations:** 1 Orthopaedics, Oceania University of Medicine, Apia, WSM; 2 Orthopaedics, Sydney Knee Specialists, Sydney, AUS

**Keywords:** total knee arthroplasty, kinematic alignment, interobserver reliability, prosthesis alignment, computed tomography (ct ), perth ct protocol, total knee arthroplasty (tka)

## Abstract

Background: Total knee arthroplasty (TKA) has become the treatment of choice for advanced osteoarthritis. Identifying malalignment is central to improving TKA outcomes and providing optimal management of TKA patients with post-operative pain and dissatisfaction. Computed tomography (CT) imaging has become increasingly popular as a more precise way of analysing post-TKA component alignment and the Perth CT protocol remains the current predominant assessment tool. This study aimed to analyse and compare inter- and intra-observer agreement of a post-operative multi-parameter quantitative CT assessment (Perth CT protocol) in TKA patients.

Methods: Post-operative CT images of 27 patients who underwent TKA were analysed retrospectively. Images were analysed by an experienced radiographer and a final-year medical student at least two weeks apart. Measurements for nine angles were collected: modified hip-knee-ankle (mHKA) angle, lateral distal femoral angle (LDFA) and medial proximal tibial angle (MPTA), femoral flexion and tibial slope, femoral rotation angle, femoral-tibial match rotational angle, tibial tubercle lateralisation distance, and Berger’s tibial rotation. Intra-observer and inter-observer intraclass correlation coefficients (ICCs) were calculated.

Results: Inter-observer reliability for the measurements of all variables varied from poor to excellent (ICC: -0.003 to 0.981). Five out of the nine angles demonstrated good to excellent reliability. Inter-observer reliability was highest for mHKA in the coronal plane and the poorest for the tibial slope angle in the sagittal plane. The intra-observer reliability for both reviewers was excellent (0.999 vs. 0.989).

Conclusion: This study demonstrates that the Perth CT protocol has excellent intra-observer reliability and good to excellent inter-observer reliability for five out of nine of the measured angles used to assess component alignment post-TKA, making it a useful tool for surgical outcome prediction and success.

## Introduction

Over the years, total knee arthroplasty (TKA) has become the treatment of choice for advanced osteoarthritis [1]. Data collection and patient surveys suggest that around 80% of patients are satisfied with their results, leaving close to 20% of patients unhappy with outcomes [2,3]. Of those requiring revisions, around 9% are a result of knee pain and poor function resulting from maltracking and prosthesis instability [4,5].

A functional and ideally pain-free total knee replacement has to be well-aligned and appropriately tracked through its range of movement. For this to be achieved, the implant components need to lie in the correct coronal, sagittal, and axial planes [5,6]. The overall outcome of TKA depends on several factors, including appropriate alignment of components, adequate soft-tissue balancing, and other patient-related factors [7,8]. Abnormal alignment in the coronal and sagittal plane has been reported to be a cause of early loosening and prosthesis failure, leading to revision surgery [9-11]. Component rotational mismatch leads to poor patella tracking, abnormal polyethylene wear, and subsequent anterior knee pain, contributing to post-TKA pain and patient dissatisfaction [12-15]. Thus, identifying malalignment is central to improving TKA outcomes and providing optimal management of TKA patients with pain and dissatisfaction post-operatively.

Historically, component alignment post-TKA was assessed using radiographs to successfully measure valgus/varus/flexion/extension angles in the coronal and sagittal planes [8,16-19]. More recently, the literature surrounding best-practice joint replacement shows CT imaging has become increasingly popular as a more precise way of analysing TKA component alignment, especially rotational alignment, and its association with post-operative issues or dissatisfaction [6,20-23].

In 2004, Chauhan et al. developed a protocol using a multi-parameter CT-based technique to assess post-operative knee alignment. The Perth CT protocol allows direct measurement of the alignment of the femoral and tibial components in relation to the mechanical axis or the trans-epicondylar axis. This technique measures all the parameters in which the components vary, namely, the alignment of the femoral and tibial prostheses in the coronal, sagittal, and axial planes, and the ways in which the femoral and tibial components are rotationally matched with respect to each other [6]. The Perth CT protocol remains the predominant assessment tool used to date for post-operative analysis of alignment, with most radiology firms deriving their scanning methodology from it, albeit with slight modifications [4,6]. This proliferation of CT scan protocols without standardisation, however, produces subsequent variations in methodology, lacks reproducibility, and makes comparison very difficult across orthopaedic practice [4,24]. Thus, despite the increasing use of the Perth CT protocol, current literature highlights a low intra-observer variability for this assessment, with inter-observer error yet to be commented on [4,6].

The aim of this study was to analyse and compare both inter- and intra-observer agreement of a post-operative multi-parameter quantitative CT assessment (Perth CT protocol) in TKA patients, namely, the alignment of the femoral and tibial prostheses in the coronal, sagittal, and axial planes and tibial and femoral component rotational matching.

It is hypothesised that there is an excellent inter- and intra-observer agreement of Perth CT protocol measurements in TKA patients.

## Materials and methods

Patients

Lower limb CT images were collected retrospectively from 27 patients who underwent robotic, kinematically aligned TKA in the private practice of three Sydney-based consultant orthopaedic surgeons (Sydney Knee Specialists) from a randomly selected period, specifically the month of April 2021 (7/4/2021 to 30/4/2021). A convenience sampling method was used. There were 15 males and 12 females. The average age at the time of the scan was 67 years with a range of 49 to 86 years.

Approval from the Hunter New England Human Research Ethics Committee was obtained. Inclusion criteria included TKA with robotically assisted and kinematically aligned surgical techniques. Unilateral/partial knee arthroplasty, mechanically aligned surgical technique, and patients unable to undergo post-operative CT scanning were excluded.

Post-operative CT image measurements (materials)

Post-operative lower limb CT imaging was performed for each patient, as per surgeon protocol. The helical scan covers from just above the acetabulum to just below the ankle joint. The field of view covers both legs. Image reconstruction used 2 mm thick slices at 1.6 mm intervals. Low doses of 2-3 mSv were used.

Measurements (method/procedure)

Two independent reviewers, an experienced radiographer (FK) and a final year medical student (LJ), measured and analysed each patient’s lower limb CT twice, more than two weeks apart, to determine inter- and intra-observer reliability. Using the Perth CT protocol as described by Chauhan et al., a total of nine measurements in three different planes for each patient were evaluated on a Siemens workstation (Munich, Germany) [6]. Measurements included the following: modified hip-knee-ankle (mHKA) angle, lateral distal femoral angle (LDFA), and medial proximal tibial angle (MPTA) in the coronal plane; femoral flexion and tibial slope in the sagittal plane; femoral rotation angle, femoral-tibial match rotational angle, tibial tubercle lateralisation distance, and Berger’s tibial rotation angle in the axial/translational plane.

Statistical analysis

Inter- and intra-observer reliability was assessed using intraclass correlation coefficients (ICCs) [25]. The calculation is based on a mean rating, absolute agreement, and two-way mixed effects model. ICC values were interpreted as follows: ≤0.5 = poor reliability; 0.51-0.75 = moderate reliability; 0.76-0.9 = good reliability; and ≥0.91 = excellent reliability [25]. All statistical analyses were performed with Microsoft Excel (Microsoft Corporation, Redmond, WA). The p-value for statistical significance was set at 0.05.

## Results

Twenty-seven patients (15 males and 12 females) with a mean age at the time of the scan of 67 (67.39 ± 8.99) years were included in the study (Table 1). The inter-observer reliability for the measurements of all variables in the three planes (coronal, sagittal, and axial) varied from poor to excellent (ICC: -0.003 to 0.981).

**Table 1:** Demographic and clinical characteristics of study participants.

Characteristics	Patients (n = 27)
Age (years) (mean ± standard deviation)	67.39 ± 8.99
Gender	15 males
	12 females
Side of total knee arthroplasty	14 right
	13 left
Surgeon	16, Surgeon 1
	8, Surgeon 2
	3, Surgeon 3

In the coronal plane, the inter-observer ICC for the mHKA angle was 0.981, indicating excellent reliability (p < 0.001); for the LDFA, the ICC was 0.846, indicating good reliability (p < 0.001); for the MTPA, the ICC was 0.920, indicating excellent reliability (p < 0.001).

In the sagittal plane, the inter-observer ICC for the femoral flexion angle was 0.523, indicating moderate reliability (p = 0.002); and for the tibial slope angle, the ICC was -0.003, indicating poor reliability (p = 0.506).

In the axial/translational plane, the inter-observer ICC for the femoral rotation angle was 0.570, indicating moderate reliability (p = 0.001); for the femoral-tibial match rotational angle, the ICC was 0.810, indicating good reliability (p < 0.001); the tibial tubercle lateralisation distance ICC was 0.872, indicating good reliability (p < 0.001); and the Berger’s tibial rotation angle ICC was 0.620, indicating moderate reliability (p < 0.001).

Intra-observer reliability was excellent for both reviewers. For reviewer 1, the ICC was 0.999 (p < 0.001). For reviewer 2, the ICC was 0.989 (p < 0.001).

## Discussion

This study investigated the intra- and inter-observer reliability of Perth CT protocol assessment of component alignment following TKA.

The intra-observer reliability for the measurements of all variables across the three planes was near perfect. Both reviewers showed excellent reliability, with high ICC values.

In regards to inter-observer reliability, results varied from poor to excellent. Inter-observer reliability was highest for angle measurements in the coronal plane, with both the mKHA and MTPA having excellent reliability and the LDFA having good reliability. The angle measurements in the axial plane showed slightly less reliability, with moderate to good ICC values. Inter-observer reliability was the poorest in the sagittal plane, with the tibial angle measurements being the only result with poor reliability. Notably, the p-value was greater than 0.05, indicating this result was not statistically significant, with a lack of evidence in the current study to indicate a true difference between the raters’ results.

The variation in the reliability of measurements across the three different planes is worth consideration. Accurate and precise measurements of angles based on CT images are the foundation of utilising the Perth CT protocol as an assessment tool for TKA prostheses. It requires a degree of knowledge of certain anatomic landmarks and how to identify them on CT images [6]. It has been discussed that whilst two-dimensional (2D) CT is an accurate method for determining prosthesis alignment overall, reliable identification of anatomical landmarks can still pose a problem [23]. Several errors can occur at different levels, including an incorrect reference definition (e.g. centre of the tibial plateau or ankle) and individual variability in the subjects pertaining to tibial plateau shape and radiological reference landmarks (e.g. locating the tibial base plate) [26]. Each of these could influence the images and result in greater variability in measurements.

Additionally, the clear visualisation of both the tibial base plate and the sagittal tibial anatomical axis on the same CT slice is dependent on the orientation of the patient's legs at the time of the CT scan [23,27]. The inability to find a single slice with both landmarks clearly visualised required two separate slices, each with a clear image of one of the landmarks, to be simultaneously analysed and measured using synchronized measure features. The variation in which two slices were being analysed could have an impact on the reliability of the sagittal plane angle measurements. Interestingly, when analysing the precision of identifying bony knee landmarks on CT imaging, Victor et al. also described greater inter-observer error in relation to identifying the tibial knee centre, a landmark that is vital to finding the sagittal tibial anatomical axis and, consequently, the tibial slope of the TKA [26].

Inter-observer variability has been described in previous studies. Konigsberg et al. reported similar findings to this study, with inter-observer reliability of 2D CT scan images post-TKA varying from poor to good. The intra-observer reliability was slightly higher, averaging good to very good, demonstrating similar improvements as this study when comparing inter- and intra-observer variability [24]. Of note, that study measured only two variables of component alignment, namely, the femoral and tibial rotation, and utilised the protocol established by Berger et al. [13], which varies slightly from the Perth CT protocol, although it was a known influence in establishing the protocol. However, when comparing the two specific angles of this study that correlate with those of Konigsberg et al.'s study, some similarities are noted.

The inter-observer reliability for tibial rotation in this study was moderate, with an ICC value of 0.620. Similarly, Konigsberg et al. found the overall ICC measurement for inter-observer reliability was 0.67 for tibial rotation. Despite slightly varied ICC classification categories, the ICC values for inter-observer reliability for tibial angle were comparable across both studies. The femoral rotation angle comparison was less alike. The inter-observer reliability for femoral rotation in this study was moderate with an ICC value of 0.570. For the same measured angle, femoral rotation in the axial plane, Konigsberg et al. reported an overall ICC value of 0.368, indicating poor reliability [24]. Both studies described a similar process to measuring the femoral rotation angles, namely, comparing the epicondylar axis with the posterior condylar surface of the component. Again, anatomical landmark references and identification could play a role in the variation of reliability across the two studies. Additionally, Konigsberg et al. compared the measurements of three reviewers, compared with two reviewers in this study.

Despite variations of inter-observer reliability, five of the nine angles had ICC values demonstrating good to excellent reliability.

Furthermore, the intra-observer reliability for both reviewers was excellent. These findings have clinical significance, as literature continues to report the importance of proper prosthesis alignment post-TKA. Poor rotational alignment affects patellar tracking and is a cause of anterior knee pain, whilst malalignment in the coronal plane leads to early loosening. Longstaff et al. demonstrated that good sagittal, coronal, and femoral rotational alignment leads to better function post-operatively, reaching rehabilitation goals quicker and shortening hospital stays [8].

Chauhan et al. established the Perth CT protocol as a helpful and thorough means to assess the adequacy of component alignment post-operatively [6]. Specifically, the Perth CT protocol allows better analysis of rotational component alignment than historically utilised methods of radiographs [23]. Of the axial measurements, the femoral-tibial rotation match demonstrated good inter-observer reliability. Suchowersky et al. propose that of all rotation angle measurements, the femoral-tibial rotation match is of greater importance and deserves more emphasis, meaning good reliability of this angle has significance. Additionally, Longstaff et al. reported significant delays in hospital stay post-TKA for patients with high axial mismatch [8]. Thus, the findings of good to excellent reliability across both inter- and intra-observer measurements in this study support the use of the Perth CT protocol as an assessment tool of component alignment post-TKA to improve patient outcomes and functional recovery.

There are several limitations to this study worth considering. Firstly, there are currently no standardised protocols with well-defined steps and reference values or grading systems that exist for post-TKA CT imaging [4]. Berger et al. originally attempted to establish a system for assessing femoral and tibial component rotation following TKA using CT imaging as a way to identify potential factors contributing to complications and poor outcomes post-operatively [13]. This study became the precursor to an imaging protocol established by Chauhan et al., later formalised as the Perth CT protocol, that measured nine variables in three planes to determine component alignment [6]. Although utilised across radiology firms across Australia as a means of assessing surgical outcomes and problematic knees, a lack of a clearly standardised protocol can diminish reproducibility amongst different evaluators. Different radiology firms have independently amended the Perth CT protocol for their personal use, leading to irregularities in how specific measurements are taken, what constitutes a value within a “normal range”, and how that applies to functional outcomes or prosthetic longevity [4].

Furthermore, a lack of a formal standardised protocol limits the ability of these results to be generalised to populations of similar characteristics, as it allows for individual manipulation of identifying measurement landmarks and measurement processes. It is possible that these measurements are significantly different from others and a known control value would identify this bias. Whilst there is little doubt that poorly placed prostheses cause post-operative complications, a lack of formalised and reproducible CT protocol to measure TKA component alignment limits CT imaging assessment and comparison in wider orthopaedic practice and warrants further study.

Roper et al. proposed a method of three-dimensional (3D) CT scan analysis to attempt to eliminate some errors in anatomical landmark identification and selection present with the use of 2D-CT images [28]. High inter- and intra-observer reliability was reported when measuring tibial rotation using 3D-CT imaging. Although this only takes into account one of the nine variables identified in the Perth CT protocol, 3D-CT imaging may be a more reliable method of reproducing results. Certainly, Hirschmann et al. also reported high inter- and intra-observer reliability of all variables in the coronal, sagittal, and axial planes when using 3D-CT imaging [23]. Based on their findings, they recommend 3D scans for determining component orientation yet they do acknowledge that 3D reconstructed CT images do not solve the problem of having reliable anatomical landmarks for orientation.

Secondly, this is a relatively small sample size (27 knees), and only two reviewers were used. Noted above, results for the tibial angle measurement did not achieve statistical significance. The small sample size (n) may have resulted in a type II error, that is, failing to conclude there was excellent reliability when there actually was. Thus, this study may have not had enough statistical power to detect any true differences for the tibial angle parameter. Larger studies with more patients and reviewers would help to reduce sampling error and increase power. It would also help to ascertain how well the findings of this study might generalise to others, and thus, how significant these findings are to orthopaedic practice as a whole.

## Conclusions

In conclusion, this study demonstrates that the Perth CT protocol frequently used to assess component alignment post-TKA has excellent intra-observer reliability and good to excellent inter-observer reliability for five out of nine of the measured angles across the three planes. The poor reliability of the tibial angle in the sagittal plane remains worthy of consideration and highlights the need for further studies. Future research should include an investigation of this research question with more scans and more reviewers, as the resulting data would be more robust than the current study was able to elicit. Overall, the findings highlight the potential for the Perth CT protocol to be utilised as an effective and reliable assessment tool for the purpose of describing the position and orientation of prostheses components in the coronal and axial planes post-TKA. As such, this imaging technique could be very useful as a predictor of long-term surgical success and patient satisfaction.
